# An Analysis of Differentially Expressed Coding and Long Non-Coding RNAs in Multiple Models of Skeletal Muscle Atrophy

**DOI:** 10.3390/ijms22052558

**Published:** 2021-03-04

**Authors:** Keisuke Hitachi, Masashi Nakatani, Yuri Kiyofuji, Hidehito Inagaki, Hiroki Kurahashi, Kunihiro Tsuchida

**Affiliations:** 1 Division for Therapies against Intractable Diseases, Institute for Comprehensive Medical Science (ICMS), Fujita Health University, Toyoake 470-1192, Japan; hkeisuke@fujita-hu.ac.jp (K.H.); nakatani@seijoh-u.ac.jp (M.N.); nanbyou@fujita-hu.ac.jp (Y.K.); 2 Faculty of Rehabilitation and Care, Seijoh University, Tokai 476-0014, Japan; 3 Genome and Transcriptome Analysis Center, Fujita Health University, Toyoake 470-1192, Japan; hinagaki@fujita-hu.ac.jp (H.I.); kura@fujita-hu.ac.jp (H.K.); 4 Division of Molecular Genetics, Institute for Comprehensive Medical Science (ICMS), Fujita Health University, Toyoake 470-1192, Japan

**Keywords:** long non-coding RNA, mRNA, skeletal muscle atrophy, RNA-sequencing

## Abstract

The loss of skeletal muscle mass (muscle atrophy or wasting) caused by aging, diseases, and injury decreases quality of life, survival rates, and healthy life expectancy in humans. Although long non-coding RNAs (lncRNAs) have been implicated in skeletal muscle formation and differentiation, their precise roles in muscle atrophy remain unclear. In this study, we used RNA-sequencing (RNA-Seq) to examine changes in the expression of lncRNAs in four muscle atrophy conditions (denervation, casting, fasting, and cancer cachexia) in mice. We successfully identified 33 annotated lncRNAs and 18 novel lncRNAs with common expression changes in all four muscle atrophy conditions. Furthermore, an analysis of lncRNA–mRNA correlations revealed that several lncRNAs affected small molecule biosynthetic processes during muscle atrophy. These results provide novel insights into the lncRNA-mediated regulatory mechanism underlying muscle atrophy and may be useful for the identification of promising therapeutic targets.

## 1. Introduction

In the last decade, increasing evidence has indicated that long non-coding RNAs (lncRNAs), defined as having more than 200 bases in length, have important roles in cell proliferation and differentiation, development, and the maintenance of homeostasis via interactions with essential proteins [1]. According to the most recent update of the LncBook database, there are 268,848 lncRNA genes in humans [2]. This is more than ten times the 19,972 protein-coding genes in the GENCODE database [3] and more than an order of magnitude above the number of known microRNAs [4], which are small non-coding RNAs of 16–28 bases in length. In addition, lncRNA mutations and dysregulation are associated with the development and progression of human diseases, including cancer, cardiovascular diseases, and neurodegenerative diseases [5,6,7].

Skeletal muscle is a mechanically pivotal organ in humans; it is required for movement of body parts and the body as a whole. Skeletal muscle is also a highly plastic organ, and its mass decreases through aging, immobilization, malnutrition, and diseases, including cancer, cardiovascular diseases, and neurodegenerative diseases [8,9,10,11,12,13]. This decrease in muscle mass is referred to as skeletal muscle atrophy and is a cause of decreased activities of daily living and increased mortality from diseases. Muscle atrophy is induced by myostatin, NF-κβ, and glucocorticoid signaling, which activate the ubiquitin-proteasome and autophagy-lysosome systems to increase muscle protein breakdown [14,15,16]. In contrast, insulin-like growth factor-1 (IGF-1)/Akt/mammalian target of rapamycin (mTOR) and β-adrenergic pathways inhibit muscle atrophy by promoting muscle protein synthesis [17]. The recent discoveries of microRNAs have provided novel insights into the regulation of skeletal muscle mass [18]. In addition, several lncRNAs associated with muscle atrophy have been identified. For example, increased expression of the lncRNA *Atrolnc-1* in catabolic conditions promotes muscle atrophy by increasing NF-κB activity [19]. In a mouse model of amyotrophic lateral sclerosis, the lncRNA *Pvt1* is associated with muscle atrophy [20]. We have also found that *Myoparr*, which is expressed from the promoter region of the *myogenin* gene, promotes muscle atrophy after denervation treatment by activating myogenin expression and inhibiting BMP signaling. The inhibition of *Myoparr* attenuates the decrease in muscle mass caused by denervation [21,22]. Thus, the identification and functional characterization of novel lncRNAs associated with muscle atrophy is promising for the development of new therapies.

We have recently shown that muscle atrophy conditions can be classified into two sub-groups (disuse-mediated atrophy and systemic wasting atrophy) based on the expression levels of skeletal muscle differentiation-related lncRNAs (*DRR*, *DUM1*, *linc-MD1*, *linc-YY1*, *LncMyod*, *Neat1*, *Myoparr*, *Malat1*, and *SRA*) and genomic imprinting-related lncRNAs (*Gtl2*, *H19*, and *IG-DMR*) [23]. It is highly likely that lncRNAs showing commonly altered expression patterns in multiple muscle atrophy conditions are involved in the development or progression of muscle atrophy. However, studies of lncRNAs that are commonly altered across multiple muscle atrophy conditions are lacking [23,24]. In the present study, to identify lncRNAs involved in muscle atrophy, we performed an RNA-sequencing (RNA-Seq) analysis of lncRNAs using four different muscle atrophy models (denervation, casting, fasting, and cancer cachexia). We detected lncRNAs related to each condition as well as those showing changes of expression in all muscle atrophy conditions. A co-expression network analysis of lncRNAs and mRNAs provided further insights into the biological processes associated with muscle atrophy. These findings improve our understanding of the molecular mechanisms underlying lncRNA-mediated skeletal muscle atrophy and are expected to contribute to the development of novel therapies.

## 2. Results

### 2.1. mRNA and lncRNA Expression Changes during Skeletal Muscle Atrophy

**Figure 1:**
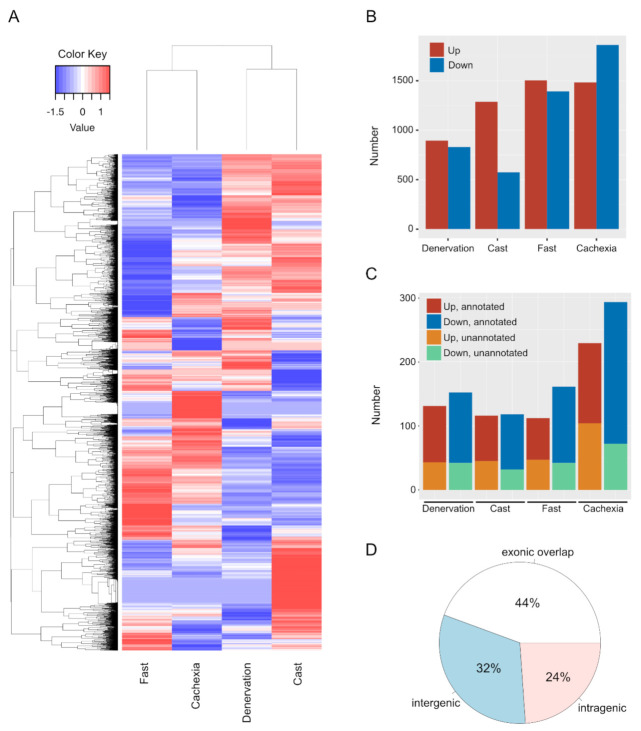
Changes in the expression levels of mRNAs and long non-coding RNAs (lncRNAs) in skeletal muscle atrophy conditions. (**A**) Hierarchical clustering analysis of differentially expressed genes, including both mRNAs and lncRNAs, after the induction of each atrophy model. (**B**) Numbers of up- and down-regulated mRNAs in each muscle atrophy condition. Red and blue indicate the up- or down-regulated mRNAs, respectively. (**C**) Numbers of up- or down-regulated lncRNAs after each treatment. Red, blue, orange, and green indicate up-regulated annotated lncRNAs, down-regulated annotated lncRNAs, up-regulated unannotated lncRNAs, and down-regulated unannotated lncRNAs, respectively. (**D**) Genomic positions of differentially expressed lncRNAs.

We performed an RNA-Seq analysis to identify protein-coding mRNAs and lncRNAs whose expression was altered across multiple skeletal muscle atrophy conditions in mice (denervation, casting, fasting, and cancer-induced cachexia). Based on the expression profiles of both mRNAs and lncRNAs revealed by RNA-Seq, muscle atrophy conditions could be classified into two sub-groups: disuse-mediated atrophy, including the denervation and casting treatments, and systemic wasting atrophy, including fasting and cancer cachexia treatments (Figure 1A). These results were consistent with the expression profiling of individual lncRNAs in our previous report [23]. We identified 895, 1288, 1505, and 1483 up-regulated and 831, 574, 1393, and 1863 down-regulated mRNAs in muscle atrophy conditions caused by denervation, casting, fasting, and cachexia treatments, respectively (Figure 1B and Table S1). In addition, we identified 88, 71, 65, and 127 up-regulated annotated lncRNAs; 110, 86, 119, and 221 down-regulated annotated lncRNAs; 43, 45, 47, and 104 up-regulated unannotated lncRNAs; and 42, 32, 42, and 72 down-regulated unannotated lncRNAs by denervation, casting, fasting, and cachexia treatments, respectively (Figure 1C and Table S1). In this study, annotated and unannotated lncRNAs were defined as sequences registered in any database and the previously unreported sequences, respectively. Forty-four percent of the identified lncRNAs showing altered expression in each muscle atrophy condition overlapped with exons of protein-coding genes or anti-sense lncRNAs against protein-coding genes (Figure 1D). Additionally, 32% and 24% of the identified lncRNAs were located in intergenic and intragenic regions, respectively (Figure 1D). Of note, all of the unannotated lncRNAs were found in intergenic regions (Table S1).

**Figure 2:**
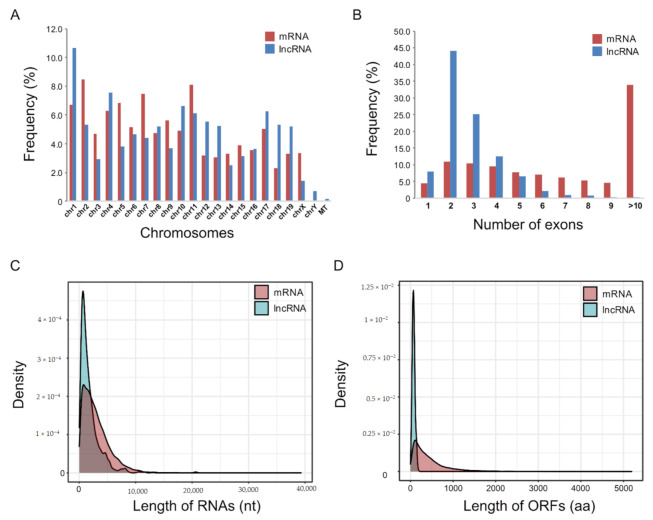
Comparison of sequence features of differentially expressed mRNAs and lncRNAs in muscle atrophy conditions. (**A**) Genome-wide distribution of differentially expressed mRNAs (red) and lncRNAs (blue) on mouse chromosomes; MT, mitochondria. (**B**) Frequency of numbers of exons in differentially expressed mRNAs and lncRNAs. (**C**) Transcript length distribution of differentially expressed mRNAs and lncRNAs; nt: nucleotide. (**D**) Distribution of open reading frame (ORF) lengths of differentially expressed mRNAs and lncRNAs; aa: amino acids.

To further characterize the identified mRNAs and lncRNAs, their structural features were examined. The chromosomal distribution of the identified mRNAs and lncRNAs did not differ significantly, except that only lncRNAs were distributed on chromosome Y and mitochondria (Figure 2A). The lncRNAs typically contained two to four exons, while more than 30% of the mRNAs contained >10 exons (Figure 2B). The mean length of lncRNAs was shorter than that of mRNAs, possibly due to the lower number of exons (Figure 2C). The lengths of the open reading frames (ORFs) of the lncRNAs were shorter than those of the mRNAs (Figure 2D).

### 2.2. Functional Characterization of Altered mRNAs in Skeletal Muscle Atrophy

**Figure 3:**
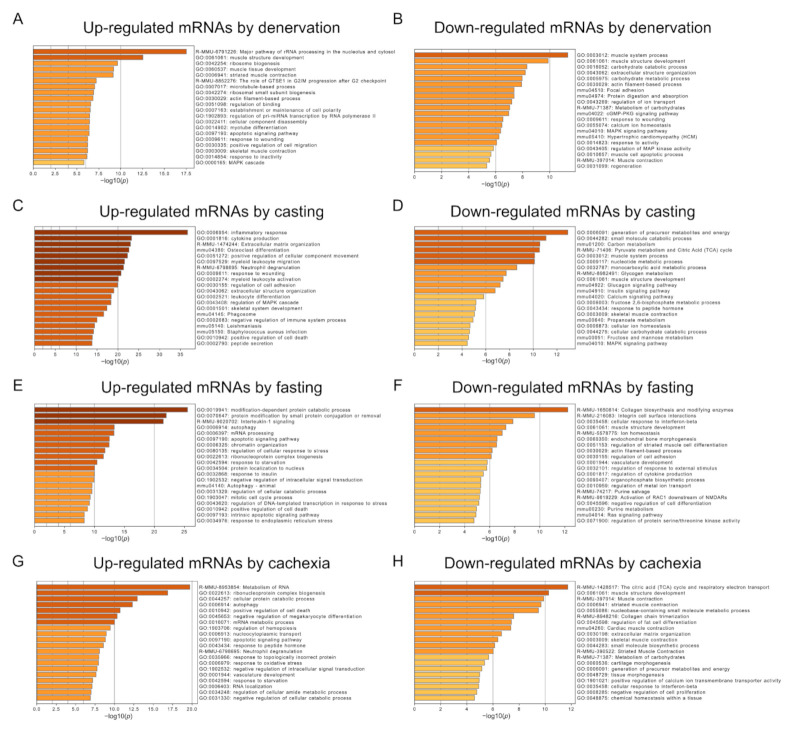
Functional enrichment analysis of differentially expressed mRNAs in each muscle atrophy condition. Enriched biological processes were ranked by *p*-value. Bar graphs show the top, non-redundant enrichment clusters (Metascape analysis) for up- or down-regulated mRNAs in response to denervation (**A**,**B**), casting (**C**,**D**), fasting (**E**,**F**), and cancer cachexia treatment (**G**,**H**). The *x*-axis represents the −log10 (*p*-value).

Next, to clarify the signaling pathways involved in each type of muscle atrophy, we examined the identified mRNAs using a functional enrichment analysis. The upregulated mRNAs, in response to denervation, were preferentially related to rRNA processing, whereas mRNAs that were down-regulated in denervated muscles were enriched in muscle system processes (Figure 3A,B). The up- or down-regulated mRNAs, after casting treatment, were enriched in the inflammatory response or generation of precursor metabolites and energy categories, respectively (Figure 3C,D). Fasting treatment increased the expression levels of mRNAs related to the modification-dependent protein catabolic processes, protein modification by small protein conjugation or removal, and interleukin-1 signaling (Figure 3E). The expression levels of mRNAs related to collagen biosynthesis and modifying enzymes decreased by fasting treatment (Figure 3F). The mRNAs upregulated by cachexia were enriched in the metabolism of RNA and ribonucleoprotein complex biogenesis categories (Figure 3G). The mRNAs involved in the citric acid cycle and respiratory electron transport were down-regulated in atrophied muscle caused by cancer cachexia (Figure 3H). These results indicated that the primary activated or repressed signaling pathways differed among the muscle atrophy conditions.

**Figure 4:**
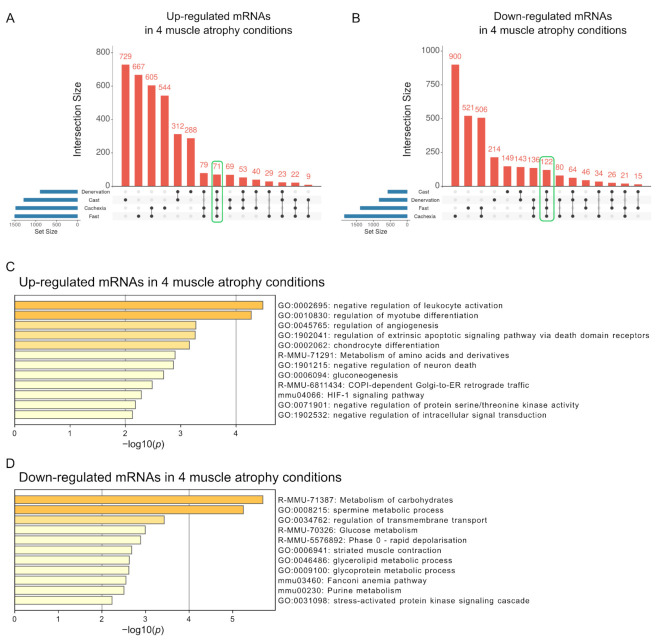
Functional enrichment analysis of mRNAs with altered expression across all four muscle atrophy conditions. The UpSet plot shows the intersections of up- (**A**) or down-regulated (**B**) mRNAs among four muscle atrophy conditions. Each column corresponds to an exclusive intersection containing the elements of the set indicated by the black circles but not the elements of any other set. Set sizes related to blue horizontal bars represent the total number of differentially expressed mRNAs for each condition. The intersection sizes between different muscle atrophy conditions represent exclusive intersections (i.e., intersection sets that are not a subset of other intersection sets). The number of mRNAs whose expression was commonly altered in all four muscle atrophy conditions is encircled by the green line. The results of functional enrichment analyses of up- (**C**) or down-regulated (**D**) mRNAs common to the four muscle atrophy conditions are shown by a bar graph generated using Metascape. The *x*-axis represents the −log10 (*p*-value).

It is likely that mRNAs with altered expression across all four muscle atrophy conditions are involved in the development or progression of skeletal muscle atrophy. We identified 71 and 122 mRNAs whose expression levels were commonly increased and decreased in all muscle atrophy conditions, respectively (Figure 4A,B and Table S2). We evaluated these common mRNAs using a functional enrichment analysis. Interestingly, upregulated mRNAs under the four muscle atrophy conditions were related to the negative regulation of leukocyte activation and regulation of myotube differentiation pathways (Figure 4C). These pathways include genes identified as negative (*Cdkn1a* and *Ctsl*) [25,26] and positive (*Abcc8*, *Csrp3*, *Dlg5*, *Gdf5*, *Gpnmb*, *Il4ra*, and *Runx1*) [27,28,29,30,31,32,33,34] regulators of muscle atrophy, supporting the validity of our analysis. Consistent with previous studies of each muscle atrophy condition [35,36,37,38,39], mRNAs with decreased expression in all muscle atrophy conditions were related to the terms of metabolism of carbohydrates and spermine metabolic processes (Figure 4D). Thus, these signaling pathways may be involved in the development or progression of skeletal muscle atrophy.

### 2.3. Identification of lncRNAs That Are Altered in Skeletal Muscle Atrophy

**Figure 5:**
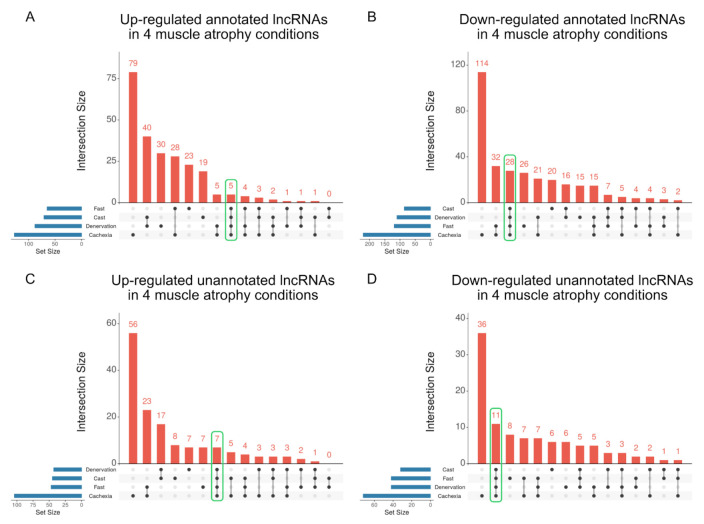
Identification of lncRNAs that are commonly altered in four muscle atrophy conditions. The UpSet plot represents the number of up- (**A**) or down-regulated (**B**) annotated lncRNAs by conditions identified by linked dots below the *x*-axis. Set sizes related to blue horizontal bars represent the total number of differentially expressed lncRNAs for each condition. The intersections represent the up- (**C**) or down-regulated (**D**) unannotated lncRNAs among muscle atrophy conditions. The number of lncRNAs that were commonly altered in all four muscle atrophy conditions is encircled by the green line.

We next extracted lncRNAs that were up- or down-regulated in all four atrophy conditions. Five and 28 annotated lncRNAs were up- and down-regulated in all muscle atrophy conditions, respectively (Figure 5A,B and Table S3). Seven unannotated lncRNAs were commonly up-regulated, and 11 unannotated lncRNAs were commonly down-regulated in all four muscle atrophy conditions, respectively (Figure 5C,D and Table S4). 

We estimated the molecular functions of identified lncRNAs by a co-expression network analysis. Correlation analysis based on Spearman’s correlation coefficients for the identified lncRNAs (33 annotated and 18 unannotated lncRNAs) and 193 mRNAs (71 up-regulated and 122 down-regulated mRNAs) and following functional enrichment analysis of correlated mRNAs showed that the expression changes of four identified lncRNAs, including down-regulated annotated lncRNAs (*linc-Myh* (*2310065F04Rik*) and *Gm49794*) and down-regulated unannotated lncRNAs (*G408* and *G10427*), were related to changes in mRNAs involved in small molecule biosynthetic processes (Figure S1A). The lncRNA–mRNA co-expression networks also indicated that decreased expression levels of the above lncRNAs affect small molecule biosynthetic processes during muscle atrophy (Figure S1B,C).

**Figure 6:**
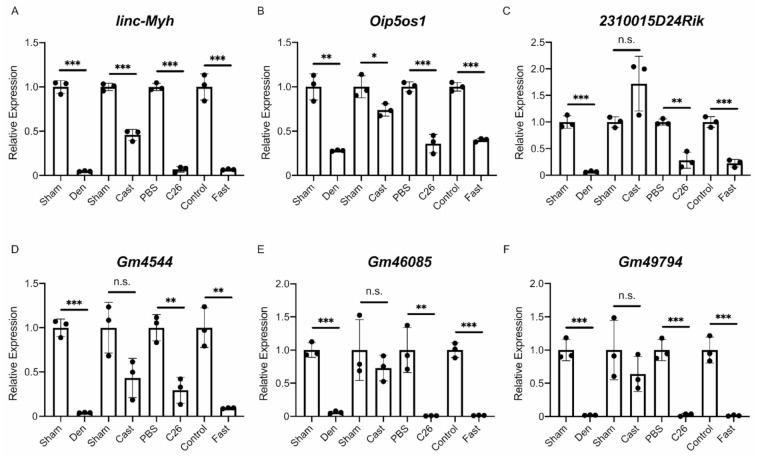
Quantification of six differentially expressed annotated lncRNAs. (**A**–**E**) The quantitative RT-PCR (qRT-PCR) results for the expression of *linc-Myh* (**A**), *Oip5os1* (**B**), *2310015D24Rik* (**C**), *Gm4544* (**D**), *Gm46085* (**E**), and *Gm49794* (**F**) in four muscle atrophy conditions. Sham; sham-operated tibialis anterior (TA) muscles. Den; denervated TA muscles. Cast; casting-operated TA muscles. PBS; TA muscles of phosphate-buffered saline (PBS)-injected control mice. C26; TA muscles of C26 tumor-bearing mice. Control; TA muscles of mice provided water and food ad libitum. Fast; TA muscles of fasting-treated mice. Data were normalized to *Rpl26* expression and are shown as relative expression. *n* = 3 biological replicates per group, mean ± SD. Dots represent the value of each sample. * *p* < 0.05; ** *p* < 0.01; *** *p* < 0.001; *n*.*s*.: not significant.

**Figure 7:**
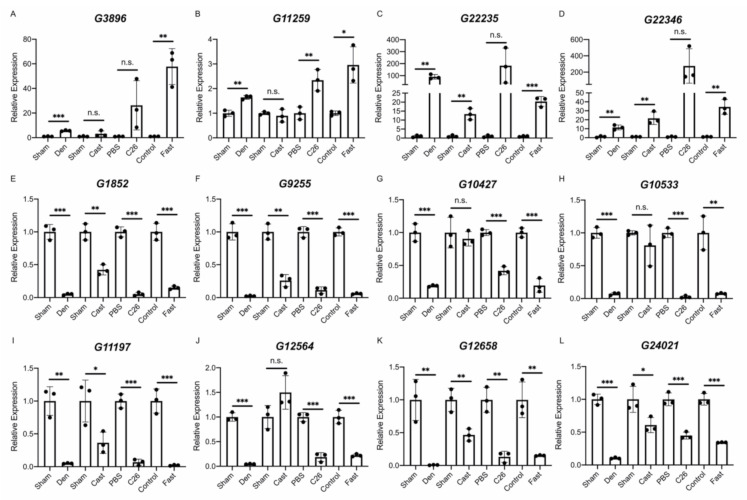
Changes in the expression of unannotated lncRNAs in four muscle atrophy conditions. (**A**–**L**) The qRT-PCR results for *G3896* (**A**), *G11259* (**B**), *G22235* (**C**), *G22346* (**D**), *G1852* (**E**), *G9255* (**F**), *G10427* (**G**), *G10533* (**H**), *G11197* (**I**), *G12564* (**J**), *G12658* (**K**), and *G24021* (**L**) in four muscle atrophy conditions. Data were normalized to *Rpl26* expression and are shown as relative expression. *n* = 3 per group, mean ± SD. Dots represent the value of each sample. * *p* < 0.05; ** *p* < 0.01; *** *p* < 0.001; *n*.*s*, not significant.

Finally, the results of the RNA-Seq analysis were validated by quantitative PCR (qPCR). We performed a quantitative analysis of multiple identified lncRNAs, including the four lncRNAs extracted from the lncRNA–mRNA network analysis. It should be noted that *G408*, identified as an unannotated lncRNA in this study, was identical to the lncRNA *Gm46085* based on a detailed sequence analysis. Therefore, *G408*/*Gm46085* was regarded as an annotated lncRNA in the qPCR analysis. Six annotated lncRNAs (*linc-Myh*, *Oip5os1*, *2310015D24Rik*, *Gm4544*, *Gm46085*, and *Gm49794*) were evaluated in each muscle atrophy condition. The quantification results obtained by qPCR were almost identical to the results of the RNA-Seq analysis except for the expression of *2310015D24Rik* in the casting model (Figure 6A–F). For unannotated lncRNAs, in addition to *G10427*, 11 lncRNAs were randomly selected from identified unannotated lncRNAs that were commonly up- or down-regulated in all muscle atrophy conditions. The qPCR results for the up-regulated unannotated lncRNAs were generally consistent with the RNA-Seq results, although some differences were not statistically significant (Figure 7A–D). The expression levels of the eight down-regulated unannotated lncRNAs decreased significantly in all atrophy conditions except for the casting samples (Figure 7E–L). These results suggest that the lncRNAs identified by our RNA-Seq analysis are generally involved in the development or progression of skeletal muscle atrophy.

## 3. Discussion

Recent global transcriptome analyses have identified numerous lncRNAs involved in the regulation of skeletal muscle formation and differentiation, indicating the importance of lncRNAs in myogenesis [24]. However, little is known about the functions of lncRNAs in the regulation of skeletal muscle mass. We have previously examined the expression levels of 12 annotated lncRNAs in six muscle atrophy conditions [23]. Although these lncRNAs were up- or down-regulated in each muscle atrophy condition, none of them showed consistent expression changes across all muscle atrophy conditions. In this study, we performed a transcriptome analysis using four muscle atrophy models (denervation, casting, fasting, and cancer-induced cachexia) and found thousands of lncRNAs that were differentially expressed in each muscle atrophy. One-third of these lncRNAs were unannotated lncRNAs in intergenic regions, indicating that RNA-Seq could be a powerful tool not only for revealing the expression changes of known lncRNAs but also for identifying novel lncRNAs associated with muscle atrophy. Furthermore, we identified 33 annotated and 18 unannotated lncRNAs that showed altered expression across four muscle atrophy conditions. A quantitative PCR analysis of 18 lncRNAs randomly chosen from the total set of 51 lncRNAs confirmed the RNA-Seq results. Taken together, we identified 51 lncRNAs associated with muscle atrophy, providing candidates for further functional analyses to elucidate the molecular mechanisms underlying the development or progression of skeletal muscle atrophy.

Molecular functions for most of the lncRNAs identified in this study are still unknown. However, the expression levels of *Oip5os1* and *linc-Myh* were substantially decreased in all muscle atrophy conditions. *Oip5os1*, also known as *OIP5-AS1*/*cyrano*, was initially implicated in the development of the brain and eye by morpholino studies in zebrafish [40]. However, subsequent studies have shown that *Oip5os1* mutant mice and zebrafish exhibited normal development [41]. More recently, Yang et al. found that *Oip5os1* contributed to myogenesis [42]. After myogenic differentiation, increased *Oip5os1* expression increased the production of Mef2c protein, a myogenic transcription factor, by recruiting HuR to the 3′utr of *Mef2c* mRNA [42]. We observed that *Mef2c* mRNA levels decreased significantly after fasting and cachexia (Table S1). Mef2 was positively related to muscle mass in adult skeletal muscle [43]; therefore, *Oip5os1* may be involved in the regulation of muscle mass via Mef2 in muscle atrophy conditions. *Linc-Myh* is located in the *Myh3*-*Myh13* gene cluster, and a previous study has shown that the knockdown of *linc-Myh* increased the expression levels of slow skeletal muscle fiber genes, suggesting that *linc-Myh* is involved in muscle fiber-type specification in mice [44]. However, Schutt et al. recently generated *linc-Myh*-knockout mice and found no change in the muscle fiber- type distribution and instead observed muscle hypertrophy [45]. Considering the fact that *linc-Myh* restricts the proliferation of satellite cells (adults muscle stem cells) via the INO80 chromatin remodeling complex [45], an increased number of satellite cells would induce muscle hypertrophy in *linc-Myh-*knockout mice. Further analyses will reveal the precise role of the decreased expression of *linc-Myh* in muscle atrophy. Intriguingly, in our co-expression network analysis of lncRNAs and mRNAs, we identified four lncRNAs, *linc-Myh*, *Gm48065*, *Gm49794*, and *G10427*, that were involved in small molecule biosynthetic processes. Although the exact role of this pathway in the regulation of skeletal muscle mass remains unclear, it is noteworthy that *Slc39a14*, *Mt1*, and *Mt2* in this pathway are associated with muscle mass [46,47]. *Slc39a14* (*Zip14*) encodes a zinc transporter, and its expression in skeletal muscle tissues is highly activated in response to lipopolysaccharide-induced inflammation. In mice, *Slc39a14* knockout leads to muscle atrophy and metabolic endotoxemia, indicating that this gene is required to maintain skeletal muscle mass [46]. However, knockout mice of both *Mt1* and *Mt2*, which encode metallothioneins, show muscle hypertrophy with increased muscle strength via the activation of the IGF-1/Akt/mTOR pathway [47]. Therefore, lncRNAs are associated with the development and progression of muscle atrophy via the regulation of small molecule biosynthetic processes.

Skeletal muscle atrophy is the main physiological change common to all four models used in this study. However, the four models also show differences in muscle fiber-type, stem cell activity, and mitochondrial function. For example, denervation and casting induce a slow-to-fast fiber-type shift, whereas cancer cachexia and fasting cause a fast-to-slow fiber-type shift [48]. In addition, denervation and cancer cachexia increase the activity of satellite cells [49,50], and casting and fasting decrease the number of satellite cells [51,52]. Recently, Li et al. found that the lncRNA *MSTRG.42019* was associated with skeletal muscle fiber-type switching in pigs [53]. Several lncRNAs, such as *LncMyoD*, *SAM*, *H19*, and *CTTN-IT1*, affect the activity of satellite cells in mice, pigs, and sheep [54,55,56,57]. The in vivo silencing of *Pvt1* results in increases in the size and number of mitochondria [20]. However, the identification of additional novel lncRNAs is needed to elucidate the physiological roles of lncRNAs in fiber-type shifting, satellite cell activity, and mitochondrial function in skeletal muscle. Our data, obtained by optimizing the comparisons (for example, comparing denervation and casting treatments with cancer cachexia and fasting treatments to identify lncRNAs associated with fiber-type switching), not only provide candidate therapeutic targets for human muscle atrophy but also provide insights into the roles of lncRNAs in skeletal muscle physiology.

## 4. Materials and Methods

### 4.1. Animal Experiments

Mice (C57BL/6J and CD2F1) were purchased from the Japan SLC and Charles River Laboratories Japan. Mice were housed in cages with a constant temperature (24 °C) and a 12 h light: 12 h dark cycle and were provided water and food ad libitum. During the fasting experiments, no food intake was allowed. C57BL/6J mice were used for denervation, atrophy by casting and fasting, and CD2F1 mice were used for cachexia treatment. The detailed protocols for each muscle atrophy experiment and the changes in the skeletal muscle weight were described previously [23].

### 4.2. RNA-Seq Library Construction, Sequencing, and Data Analysis

RNA-Seq libraries were prepared as described previously [22]. In brief, after the induction of muscle atrophy, total RNA isolated from the tibialis anterior muscles was purified using the miRNeasy Mini Kit (QIAGEN, Hilden, Germany) with DNase I (QIAGEN, Hilden, Germany), according to the manufacturer’s protocol. One microgram of total RNA was used for the purification of Poly(A)+ RNAs using the NEBNext Poly(A) mRNA Magnetic Isolation Module (New England Biolabs, Ipswich, MA, USA). The RNA-Seq libraries were constructed using the NEBNext Ultra RNA Library Prep Kit for Illumina (New England Biolabs, Ipswich, MA, USA), according to the manufacturer’s protocol. The libraries were sequenced to obtain 124-bp single-end reads for each sample (two biological replicates per sample) using an Illumina HiSeq 1500. bcl2fastq 1.8.4 was used for base-calling. Raw sequence data were deposited into the DNA Data Bank of Japan (DDBJ; Accession IDs DRR238781 to DRR238792, Table S5). To analyze the expression profiles of mRNAs and lncRNAs in cachexia samples, published RNA-Seq data (GSE65936) [58] were used.

Raw sequence data were quality-trimmed using FastQC ver. 0.11.3 (https://www.bioinformatics.babraham.ac.uk/projects/fastqc/ 26 February 2021) with the command “-Q 33 -t 20 l 30.” The reads were aligned to the GENCODE GRCm38_p6_genome using Hisat2 v. 2.2.0 [59] with default parameters. Mapped reads were converted to Bam files using SAMtools v. 1.3.1 [60]. Read counts were obtained using StringTie v. 2.1.3b [59] and taco v. 0.7.3 [61] from the Bam files using the gencode_vM25_Annotation.gtf file with default parameters. Gene_count_matrix data produced by read counting were used for statistical analyses of differentially expressed genes by DESeq2 v. 1.26.0 [62] with the Wald test (cut-offs: base-mean > 10, false discovery rate (adjusted *p*-value, padj) < 0.05, and log2 fold change of >1 or <−1).

The protein-coding potential was calculated by the Coding Potential Assessment Tool (CPAT) [63]. In this study, lncRNAs were defined as RNAs categorized as lncRNAs by CPAT with no CPAT coding label. mRNAs and lncRNAs were counted on the basis of the gene_id. The transcript_id was used to examine the chromosomal location, number of exons, RNA length, and ORF size. An enrichment analysis was performed using Metascape [64]. The lncRNA–mRNA network was analyzed by calculating correlation coefficients from normalized read count data for lncRNAs and mRNAs whose expression levels were altered in all muscle atrophy conditions. According to a previous report [65], Spearman’s correlation coefficients > 0.9 (positive) or <−0.9 (negative) and a value of *p* < 0.05 were considered significant. After an enrichment analysis of extracted mRNAs by Metascape, the co-expression network between lncRNAs and mRNAs related to small molecule biosynthetic processes was visualized by Cytoscape [66].

### 4.3. RNA Purification, Reverse Transcription, and qPCR

One microgram of total RNA was used to generate cDNAs by reverse transcription using Protoscript II Reverse Transcriptase (New England Biolabs, Ipswich, MA, USA) with random primers. Quantitative PCR was performed using SYBR Premix Ex Taq (Takara, Kusatsu, Japan). Relative gene expression was determined using a ΔΔCt method and normalized to a reference gene (*Rpl26* expression). Values are shown as each control (sham-operated muscles, phosphate-buffered saline (PBS)-injected control mice, or muscles of mice provided water and food ad libitum) is 1. The primers are listed in Table 1. Statistical analyses were performed using unpaired, two-tailed Student’s *t-*tests. A value of *p* < 0.05 was considered statistically significant.

## Figures and Tables

**Table 1:** Primer sequences used for quantitative PCR.

Target Name	Forward	Reverse
*linc-Myh*	GTGCAGCCAGAACAAGACAG	CAAGATGGGAGGCTCTCAAA
*Oip5os1*	ATAAACAGGCGCCACCATCA	CAGCACAGCCTGAGTCTGAA
*2310015D24Rik*	AGCTATCCACAGCCAGAGGA	GACGTCAAAGGTCTGCAGGA
*Gm4544*	CTGACTCCCCCAAGTTGTCC	GAGCTGTGATTGCAGATGCG
*Gm46085*	AAACCCCCAAAACCCCAACT	CCCTGGGTCCTCATTTTCCC
*Gm49794*	GTCAACTGCCTTAGCCAGGT	TCACAGCTTCTGCACCTCAG
*G3896*	AGAGGAGGCAGGGTAACGAT	CCGTGGGTTCTGCTTTTTGG
*G11259*	AAACCGTACCACTGGAGCAG	CAGTTTCCCTATGCAGCCCA
*G22235*	TTTTCCCAGTGCCCAACAGT	ACACTGAATACCCTGGCTGC
*G22346*	TTTCTGTGACTCCGTGACCG	TTGCAAGGAGATGGCGTTCT
*G1852*	TTGCCATCACCAGTAGCCTG	GGGAGTGGCTCTCTCAGAGA
*G9255*	AATCCTCTCCCCAGAGCTGT	AGGAGGACCAATACCCAGGG
*G10427*	CCACCTTTGACTCAGGCCTT	TATGATGAGCTGGGACCCCA
*G10533*	TCGAGTCTGTTCCACATGGC	TCCTCAGGGCTAGGACTTCC
*G11197*	AAGAGAGTGTTTCCTGGCCG	TGAGGGATTGCATGTGCCAT
*G12564*	GCCTGAGGACATTGTGGTGA	TGTCTTCTTCAGGCAGCCAG
*G12658*	GCAGAGACCTTTCTGACCCC	GGTGGTGACAGAGAAGGGTG
*G24021*	GTTCCTGTACCTGGGGTTGG	ACATGTGGGTGAGCTGATGG
*Rpl26*	GGTCTATGCCCATTCGGAAGG	TCGTTCGATGTAGATGACGTACT

## Data Availability

The RNA-seq raw data for each sample reported in this study were deposited into the DDBJ Sequence Read Archive under the accession No. DRA010545.

## Supplementary Materials

The following are available online at https://www.mdpi.com/1422-0067/22/5/2558/s1, Figure S1: Co-expression network for lncRNAs and mRNAs whose expression levels were altered in all atrophy conditions, Table S1: Up- or down-regulated mRNAs and lncRNAs in each muscle atrophy condition, Table S2: Up- or down-regulated mRNAs across four muscle atrophy conditions, Table S3: Up- or down-regulated annotated lncRNAs across four muscle atrophy conditions, Table S4: Up- or down-regulated unannotated lncRNAs across four muscle atrophy conditions, Table S5: List of raw sequence data.
