# Supplementary material for: An Analysis of Differentially Expressed Coding and Long Non-Coding RNAs in Multiple Models of Skeletal Muscle Atrophy
Source: Int J Mol Sci. 2021 Mar 4;22(5):2558. doi: 10.3390/ijms22052558 (PMC7961583; doi:10.3390/ijms22052558)
Supplement: Supplementary file 1 [file ijms-22-02558-s001.zip › supplementary files/Figure S1.docx]

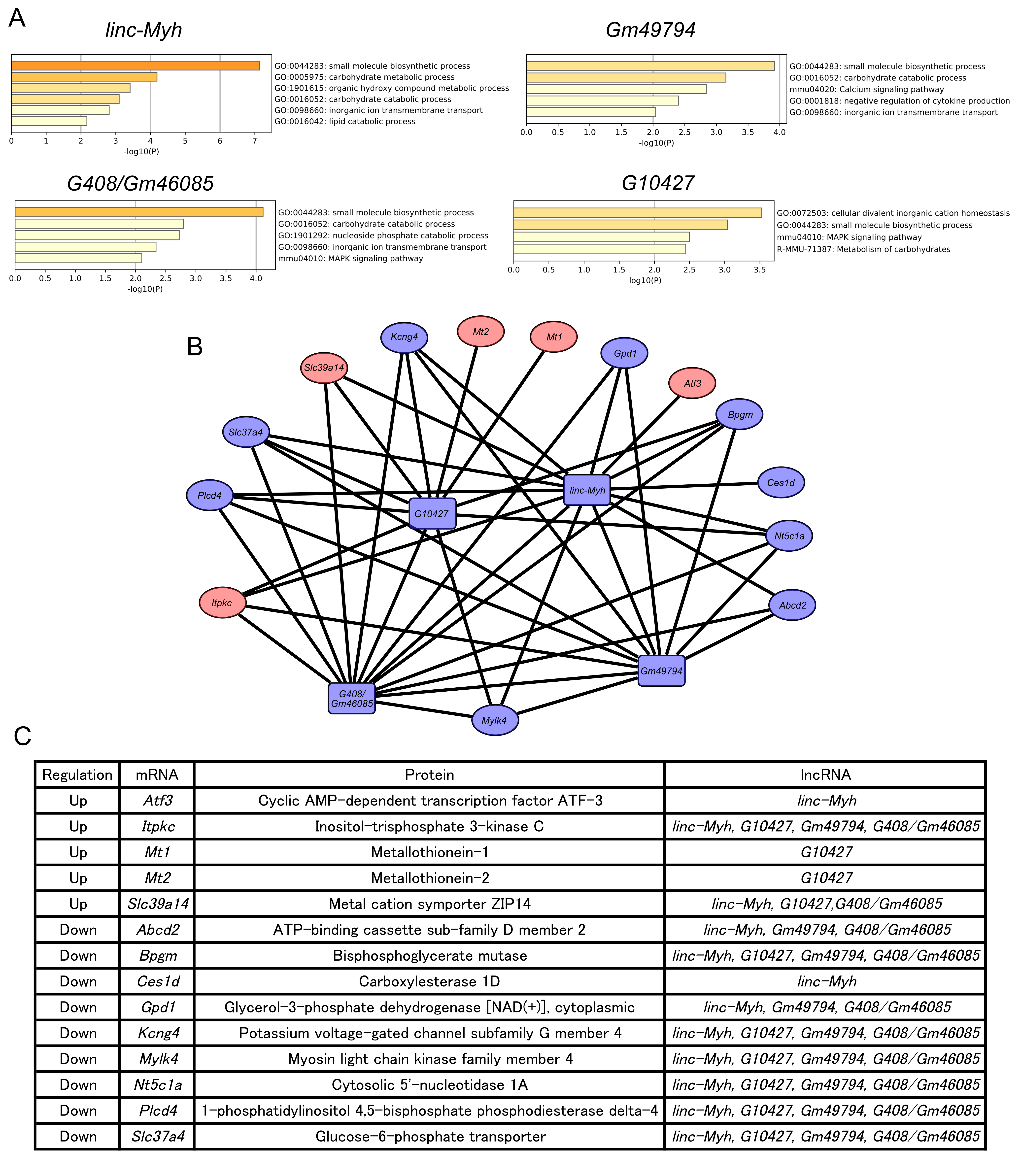
**Figure S1.** Co-expression network for lncRNAs and mRNAs whose expression levels were altered in all atrophy conditions. (A) Functional enrichment analysis of mRNAs whose expression was correlated with the expression of indicated lncRNA. Enriched biological processes were ranked by p-value. Bar graphs show the top non-redundant enrichment clusters (Metascape analysis). The *x*-axis represents the −log10 (p-value). (B) The co-expression network between lncRNAs and mRNAs related to small molecule biosynthetic processes. The red and blue circles represent the up- or down-regulated mRNAs in all muscle atrophy conditions, respectively. The blue squares mean down-regulated lncRNAs in all muscle atrophy conditions. The connection between lncRNA and mRNA means that these expressions were correlated. (C) Detailed information for mRNAs that show correlated expression with indicated lncRNAs and also are related to small molecule biosynthetic processes. The regulation indicates the direction of expression changes of mRNAs during muscle atrophy.
